# The Outcomes of Sodium-Glucose Co-transporter 2 Inhibitors (SGLT2I) on Diabetes-Associated Neuropathy: A Systematic Review and meta-Analysis

**DOI:** 10.3389/fphar.2022.926717

**Published:** 2022-07-11

**Authors:** Mahmoud Kandeel

**Affiliations:** ^1^ Department of Biomedical Sciences, College of Veterinary Medicine, King Faisal University, Al-Hofuf, Saudi Arabia; ^2^ Department of Pharmacology, Faculty of Veterinary Medicine, Kafrelshikh University, Kafrelshikh, Egypt

**Keywords:** peripheral neuropathy, diabetes, SGLT2I, meta-analysis, dapagliflozin

## Abstract

Diabetes mellitus (DM) is one of the leading causes of morbidity and mortality worldwide. DM patients with diabetic neuropathy (DN) usually present with distal pain, sensorimotor polyneuropathy, postural hypotension, or erectile dysfunction. They also may present with other nerve pathologies such as inflammatory neuropathies and carpal tunnel syndrome. We conducted a systematic review and meta-analysis to assess the benefits of using sodium–glucose co-transporter-2 inhibitors (SGLT2Is) to manage DN. An extensive systematic literature review was conducted to include all articles published up to 24 February 2022. All clinical studies included patients with DM and reported the outcomes of SGLT2I on diabetes-associated neuropathy. Six studies were identified for meta-analysis, including a total of 5312 diabetic patients. The average age of the included patients ranged from 41 to 74 years and 34–73 years in the SGLT2I treatment and control groups, respectively. SGLT2I moderately improved the manifestations of diabetic peripheral neuropathy events and nerve conduction velocity. Furthermore, the SGLT2I treatment group had a statistically significant higher mean heart-to-mediastinum ratio (MD 0.41; 95% 0.17, 0.64; *p* = 0.0006). However, the mean heart rates (MD −4.51; 95% −10.05, 1.04; *p* = 0.11) and wash out rates (MD 2.13; 95% −8.48, 12.75; *p* = 0.69) were not significantly different between the two groups. SGLT2Is could therefore be considered neuroprotective in patients with DN, possibly by considerably increasing the sensory and motor nerve conduction velocity, improving the clinical manifestations of DPN, and reducing sympathetic nervous system activity.

**Systematic Review Registration:** http://www.crd.york.ac.uk/prospero/, identifier CRD42022312828

## 1 Introduction

Diabetes mellitus (DM) leads to considerable global morbidity and mortality. The current worldwide prevalence of DM is approximately 11%, expected to increase to 13% by 2045. The disease is especially prevalent in North Africa and the Middle East (Saeedi et al., 2019). DM is associated with microvascular (peripheral vascular disease, cardiovascular disease, and stroke) and macrovascular (diabetic neuropathy [DN], nephropathy, and oculopathy) complications. The most common microvascular complication is DN, which develops in approximately 50% of diabetic patients (Zimmet, 2017; IDF, 2019). DM is characterized by peripheral insulin resistance (due to hyperglycemia) followed by compensatory hyperinsulinemia, leading to mitochondrial dysfunction, increased inflammation, endoplasmic reticulum stress, and altered energy metabolism. The same disturbances occur in some neurological disorders, leading to functional alterations in neurons and cell death (Pugazhenthi et al., 2017). Patients with DN usually present with distal pain, sensorimotor polyneuropathy, postural hypotension, or erectile dysfunction. They also may present with other nerve pathologies such as inflammatory neuropathies and carpal tunnel syndrome (Pop-Busui et al., 2017). DN impairs the patient’s quality of life by increasing the risk of foot ulcers, falls, and fractures, highlighting the need for drugs that prevent and control the condition (Boulton, 2014).

Antidiabetic medications have a significant role in controlling DM and preventing complications. These drugs might positively improve brain cell metabolism, which is thought to be of clinical importance for patients with DM-associated neurological disorders. In recent years, research has led to the considerable progression of antidiabetic drugs, including glucagon-like peptide-1 receptor agonists, dipeptidyl peptidase-4 inhibitors, and sodium–glucose co-transporter-2 inhibitors (SGLT2Is). These drugs can reduce glycated hemoglobin, do not promote hypoglycemia, and can cause weight loss. SGLT2Is reduce plasma glucose by inhibiting the reabsorption of glucose from the kidney regardless of cell mass or function, inducing glucosuria. Glucose loss in urine results in negative energy balance and weight loss, and the corresponding inhibition of sodium absorption in the proximal tubules decreases blood pressure (Vasilakou et al., 2013). SGLT2Is exert an insulin-independent hypoglycemic effect that can reduce blood glucose even with decreased insulin secretory capacity. This mechanism improves insulin resistance, decreases glucose toxicity, and enhances pancreatic cell function. SGLT2Is also improve mitochondrial function and insulin signaling in the brain and prevent cognitive decline by protecting synaptic plasticity. Furthermore, by elevating blood ketone bodies, SGLT2Is modulate the pathological pathway in neurodegenerative diseases (Sa-Nguanmoo et al., 2017; Wiciński et al., 2020; Sim et al., 2021).

Treating DN has been a significant challenge, even though various antidiabetic drugs have been proposed to control hyperglycemic status and diabetic complications. Moreover, although the additional metabolic benefits of SGLT2Is have encouraged ongoing clinical evaluation of these inhibitors for DN treatment (Donnan et al., 2019; Caparrotta et al., 2021), the body of available data has not been previously systematically reviewed. In our preliminary literature review, we found that most of the relevant studies were not designed to compare the short-term and long-term outcomes of SGLT2I administration for diabetic patients with neuropathy. The many limitations of these studies included convenience sample bias, heterogeneity, variable study endpoints, and inconsistent follow-up periods. These limitations resulted in inconsistent data, making it difficult to draw firm conclusions from the available evidence. Therefore, we conducted a systematic review and meta-analysis to reveal the beneficial role of SGLT2Is in treating DN patients. Knowledge of these benefits could help healthcare providers offer effective DN interventions.

## 2 Methods

### 2.1 Study Documentation and Registration

For this systematic review and meta-analysis, we followed the Preferred Reporting Items for Systematic Reviews and Meta-Analysis (PRISMA) criteria (Moher et al., 2009) and the recommendations of the Cochrane collaboration (Green et al., 2008) (Supplementary Table S1). The study’s methodology was documented in a protocol registered at the International Prospective Register of Systematic Reviews (http://www.crd.york.ac.uk/prospero/; registration number CRD42022312828).

### 2.2 Data Sources

An exhaustive literature search was implemented to include all articles up to 24 February 2022, using the following databases: PubMed, Web of Science (ISI), Google Scholar, Scopus, EMBASE, Clinical trials, Cochrane Collaboration, Virtual Health Library, NYAM, SIGLE, Controlled Trials, and WHO International Clinical Trials Registry Platform. There were no limitations on patients’ age, sex, ethnicity, language, race, or location.

The following keywords were used in every possible combination: “Sodium–glucose co-transporter 2 inhibitors”, “SGLT2”, “Dapagliflozin”, “Empagliflozin”, “Canagliflozin”, “Luseogliflozin”, “Ipragliflozin”, “Ertugliflozin”, “Sotagliflozin”, “Sergliflozin”, “Tofogliflozin”, “Remogliflozin”, “Neuropathy”, “Nerve”, “Nervus”, and “Nerves”. Within the thesaurus of each searched database, the search technique used controlled vocabulary phrases. To guarantee that a wide variety of relevant articles were screened, we used a combination of medical topic headings (MeSH) and text terms. The references within the retrieved studies were manually searched to identify any other unindexed literature. Cross-referencing was used until no further relevant articles were found.

### 2.3 Study Selection

DM patients in clinical investigations were included if the study reported the effects of SGLT2I on diabetes-related neuropathy. Guidelines, review papers, non-human research, case reports, comments, letters, editorials, posters, and book chapters were removed since the corresponding data could not be retrieved. Two reviewers separately screened titles, abstracts, and full-text publications for potentially relevant papers that matched the inclusion criteria. Any disagreement between the reviewers was resolved by discussion.

### 2.4 Data Extraction

The extracted parameters were study characteristics (the title of the included study, the first author’s last name, study period, study design, year of publication, and study region), demographic characteristics (sample size, gender, age, socioeconomic status, duration of diabetes, and comorbidities), and laboratory findings (lipid profile, glycemic status, liver functions, and renal functions). We extracted data related to DN events, improvement in neuropathic symptoms, and nerve conduction velocities. Two reviewers extracted the data independently into a Microsoft Excel spreadsheet. The data were extracted from graphs using WebPlotDigitizer software (Rohatgi, 2018).

### 2.5 Assessment of Bias

The risk of bias in the randomized clinical trials was assessed using the Cochrane Collaboration tool (Higgins et al., 2011). The quality of the observational studies was estimated using the National Institute of Health (NIH) quality assessment tool (National Heart et al., 2014).

### 2.6 Statistical Analysis

The weighted mean difference was used to analyze the continuous variables. Data reported as median and range values were converted to mean and standard deviation (SD) based on equations from Hozo et al. (Hozo et al., 2005). The fixed-effect model was employed if fixed population effects were assumed; otherwise, the random-effects model was utilized. Statistical heterogeneity was assessed using the Higgins *I* ^*2*^ statistic at > 50%, and the Cochrane Q (_*Χ*_^2^ test), at *p* < 0.10 (Higgins et al., 2003). Data analysis was performed using Review Manager software v. 5.4 (Cochrane Collaboration, Copenhagen, Denmark) and Comprehensive Meta-Analysis v. 3 software (Borenstein et al., 2005).

## 3 Results

### 3.1 Search Results

**FIGURE 1:**
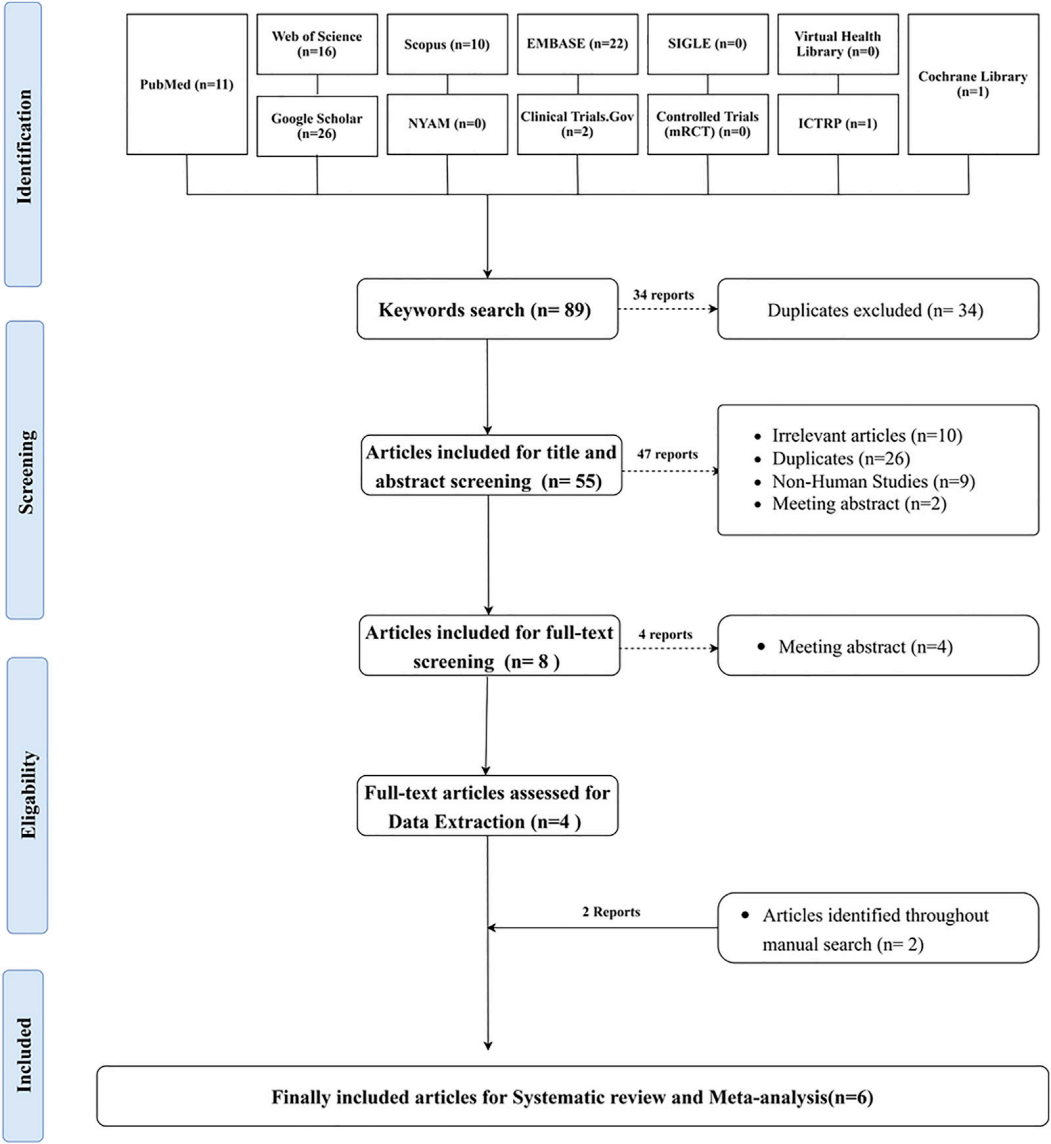
PRISMA flowchart depicting the process of doing a literature search, screening titles, abstracts, and full texts, conducting a systematic review, and conducting a meta-analysis.

The literature search yielded 89 articles, of which 34 were duplicates. Of the 55 articles included in the title and abstract screening, eight were eligible for full-text screening. Four articles were included in the data extraction process, and two additional articles were identified through the manual search. Six publications qualified for the systematic review and meta-analysis. Supplementary Table S2 shows the search technique for each database that was searched. The PRISMA flowchart depicts the searching strategy, screening, and eligibility (Figure 1).

### 3.2 Demographic Characteristics of the Included Studies

The present systematic review included six articles including 5312 diabetic patients. Four studies employed a randomized clinical design (RCT), and two studies used an observational design. The SGLT2I dose ranged from 10 to 100 mg. The average age of the included patients ranged from 41 to 74 years and 34–73 years in the SGLT2I and control groups, respectively. There were 3417 males (64.32%) with an average BMI ranging from 22.72 to 28.9 (kg/m^2^). The mean duration of DM ranged from 4.8 to 38.3 years among the SGLT2I group and from 4.3 to 32.4 years among the control group. The mean follow-up duration ranged from 6 months to 2.45 years (Table 1).

**TABLE 1:** Demographic characteristics of the included studies.

	Study ID	Study region	Study design	Study period	Intervention	Control	Sample size		Age (Years)		Gender (male)		BMI (kg/m2)		Hypertension		Duration of Diabetes		Follow-Up
							Intervention	Control	Intervention	Control	Intervention	Control	Intervention	Control	Intervention	Control	Intervention	Control	
							Number	Number	Mean ± SD	Mean ± SD	Number	Number	Mean ± SD	Mean ± SD	Number	Number	Mean ± SD	Mean ± SD	
1	Jordan et al. (2017)	Germany	RCT	13 January 2011 to 3 September 2014	empagliflozin 25 mg once daily (qd) for 5 days	Diuretic and empagliflozin in combination	22	56.0 (40–65)*	15	26.8 (23.9–29.7)	NR	NR	NR	NR	__				
2	Liao et al. (2022)	United States	RCT	19 February 2014 to 5 December 2019	Canagliflozin One 100 mg	Placebo	4401	63.0 ± 9.2	2907	31.3 ± 6.2	2172	15.8 ± 8.6	2.45 years						
3	Mehta et al. (2021)	India	RCT	September 2020 and August 2021	empagliflozin 25 mg once daily	oral hypoglycemic agent	50	50	NR	NR	29	30	26.9 ± 0.5	28.9 ± 0.7	NR	NR	NR	NR	6 months
4	Sardu et al. (2022)	Italy	Prospective case-control	June 2018 to March 2021	SGLT2I	Non SGLT2I	161	446	61 (41–74)	57 (34–73)	81	234	NR	NR	92	266	NR	NR	12 months
5	Shimizu et al. (2020)	Japan	RCT	February 2018 to March 2019	empagliflozin (10 mg/day)	Placebo	46	50	63.9 (10.4)	64.6 (11.6)	38	39	25.2 (3.7)	25.2 (4.1)	38	39	38.3 (43.4)	32.4 (43.3)	4, 12, and 24 weeks
6	Wang et al. (2019)	China	Retrospective Study	August 2017 to May 2018	mecobalamin and oral dapagliflozin (10 mg/tablet	oral mecobalamin tablets (0.5 mg/) 3 times a day	43	43	62.5 ± 5.1	63.4 ± 4.8	23	21	22.72 ± 2.08	22.65 ± 2.20	11	12	4.8 ± 2.6	4.3 ± 1.2	1 month

*Data reported in the form of mean and range, ** Data reported in the form of median and interquartile range.

RCT, Randomized controlled trial; SGLT2I, Sodium-glucose Cotransporter 2 Inhibitors, NR, Non-reported.

### 3.3 Risk of Bias and Quality Assessment

**FIGURE 2:**
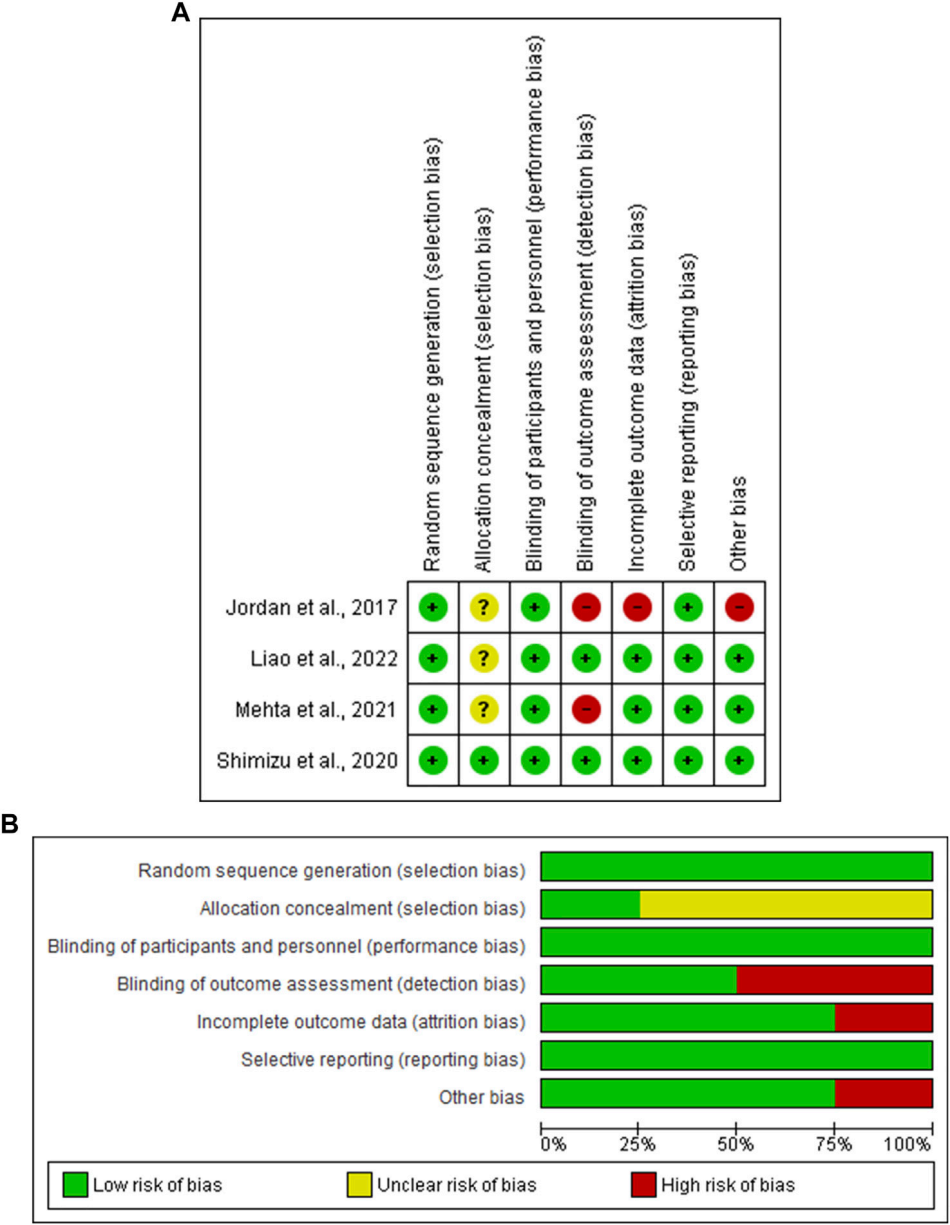
**(A)** Risk of bias graph, **(B)** Risk of bias summary: review authors’ judgments about each risk of bias item presented as percentages across all included studies.

Based on the Cochrane Collaboration’s tool for assessing the risk of bias, all four included RCTs (Jordan et al., 2017; Shimizu et al., 2020; Mehta et al., 2021; Liao et al., 2022) showed a low risk of selection bias, performance bias, and reporting bias. Three studies (Jordan et al., 2017; Mehta et al., 2021; Liao et al., 2022) showed an unclear risk of selection bias, while two (Jordan et al., 2017; Mehta et al., 2021) showed a high risk of detection bias. Three of the four studies (Shimizu et al., 2020; Mehta et al., 2021; Liao et al., 2022) showed a low risk of attribution bias, and one study (Jordan et al., 2017) showed a high risk. The NIH quality assessment tool revealed that two studies (Wang et al., 2019; Sardu et al., 2022) were good quality (Figure 2 and Table 1).

### 3.4 Outcomes of Sodium-Glucose Co-transporter 2 Inhibitors Administration

#### 3.4.1 Peripheral Neuropathy Events

Liao et al. (2022) reported the impact of SGLT2I on diabetes-associated peripheral neuropathy. Of the 4401 patients included, 334 (15.16%) experienced neuropathies in the SGLT2I group and 323 (14.68%) in the control group. Moreover, 84 (3.81%) patients experienced sensorimotor polyneuropathy events in the SGLT2I group compared to 90 (4.09%) patients in the control group. Furthermore, 84 (3.81%) and 92 (4.18%) patients experienced DN in the SGLT2I and the control groups, respectively. Wang et al. (2019) assessed the impact of dapagliflozin combined with mecobalamin on sensory and motor never conduction velocity. They reported a relative improvement in the conduction velocity of the median and common peroneal nerves in the SGLT2I group relative to patients treated with oral mecobalamin tablets only.

#### 3.4.2 Autonomic Neuropathy

##### 3.4.2.1 Heart Rate

**FIGURE 3:**
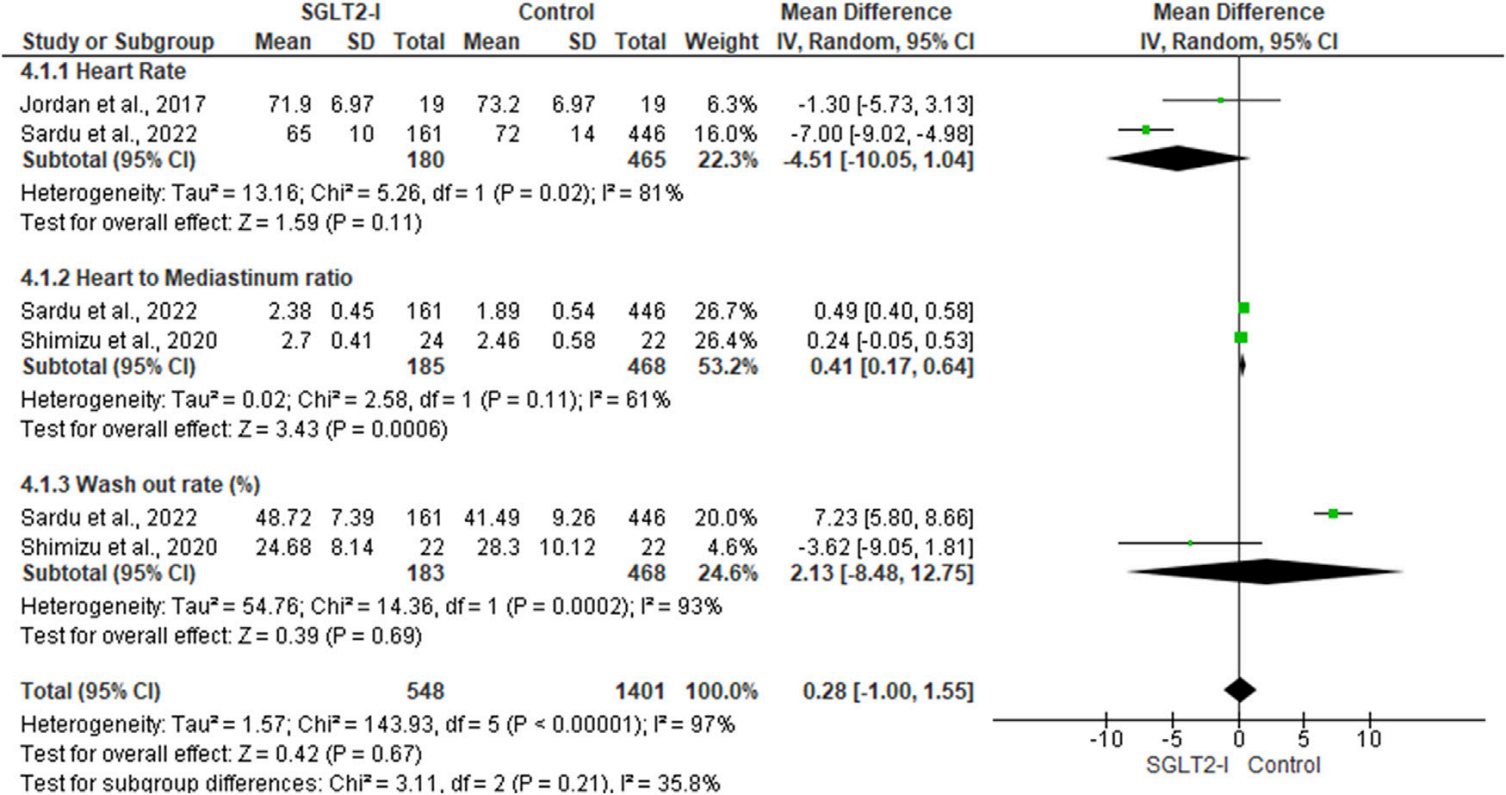
Forest plot of summary analysis of the mean difference and 95% CI of the mean heart rate between SGLT2-I and control groups; the mean heart to mediastinum ratio between SGLT2-I and control groups and the mean wash-out rate between SGLT2-I and control groups. Each trial’s statistical weight is represented by the size of the blue squares. The grey diamond represents the pooled point estimate. A significant outcome (IV = inverse variance) is indicated by the placement of diamonds and squares (together with 95 percent confidence intervals) beyond the vertical line (unit value).

Two studies (Jordan et al., 2017; Sardu et al., 2022), including 645 patients, assessed the difference in heart rate between SGLT2I and control groups. In the random-effects model (*I* ^2^ = 81%, *p *= 0.02), the difference between the two groups was not statistically significant (MD -4.51; 95% −10.05, 1.04; *p *= 0.11) (Figure 3).

##### 3.4.2.2 Heart-to-Mediastinum Ratio

Two studies (Shimizu et al., 2020; Sardu et al., 2022), including 653 patients, reported the mean heart-to-mediastinum ratio in patients treated with SGLT2I and the control group. The mean heart-to-mediastinum ratio was significantly higher in the SGLT2I group (MD 0.41; 95% 0.17, 0.64; *p* = 0.0006) in the random-effects model (*I* ^2^ = 61%, *p* = 0.11) (Figure 3).

##### 3.4.2.3 Wash out Rate (%)

The wash-out ratio difference between the SGLT2I and control groups was reported in two studies, including 651 patients (Shimizu et al., 2020; Sardu et al., 2022). In the random-effects model (*I* ^2^ = 93%, *p *= 0.0002), the two groups were not statistically different (MD 2.13; 95% −8.48, 12.75; *p *= 0.69) (Figure 3).

## 4 Discussion

Computational tools and meta-analysis are gold standards in diseases epidemiology, treatment and control (Kandeel et al., 2021; Kandeel and El-Deeb, 2022a; Kandeel and El-Deeb, 2022b; Burayk et al., 2022; Kandeel et al., 2022; Kim et al., 2022). DN is the most encountered microvascular complication of diabetes. It is treated by controlling glycemic status, improving microcirculation and the metabolic profile, and increasing neurotrophic treatments (Khdour, 2020). Although many published studies have demonstrated that SGLT2I is effective in controlling glycemic status, the effectiveness of SGLT2I for improving diabetic peripheral neuropathy warranted further investigation. The literature is still limited regarding the impact of SGLT2I on the outcomes of diabetes-associated neuropathy due to the lack of well-structured randomized clinical trials and prospective cohort studies (Lupsa and Inzucchi, 2018; El Mouhayyar et al., 2020). Therefore, this meta-analysis was performed to reveal the effectiveness of SGLT2I in improving peripheral neuropathy and autonomic neuropathy manifestations in DM patients.

The analysis revealed that SGLT2I moderately improved DPN manifestations. This includes a significant improvement in sensory and motor never conduction velocity. However, there were comparable diabetic-associated neuropathy events among the SGLT2I and the control groups. Furthermore, patients treated with SGLT2I showed a relative decline in the mean heart rate level, mean ratio of heart to the mediastinum and an increase in wash-out rate. These results imply that SGLT2I could be a promising treatment choice for patients with DPN. However, these findings should be cautiously interpreted as only differences in the heart-to-mediastinum ratio attained statistical significance.

Long-term hyperglycemia induces oxidative stress. The released oxygen-free radicals cause ischemia and hypoxia of nerve tissue, resulting in progressive cell damage. SGLT2I has been found to reduce glucose reabsorption in the renal tubules by inhibiting SGLT2 release in the proximal tubule. Furthermore, SGLT2I regulates the levels of serum malondialdehyde, superoxide dismutase, and cyclooxygenase-2, which decelerates free radical damage to nerve cells, improving DPN manifestations. Although hyperglycemia is a potential risk factor for DPN, other factors have a considerable role in DPN pathogenesis, such as obesity, hypertension, smoking, dyslipidemia, and cardiovascular diseases. These factors may explain the lack of statistical significance observed in determining the efficacy of SGLT2I in improving DPN manifestations (Tesfaye et al., 2005; Zoungas et al., 2017; van der Velde et al., 2020). Mitochondrial dysfunction and lipid metabolism disturbance contribute to the microvascular complications of DM. The treatment goal is not simply to control the glycemic status but to pursue novel therapies targeting these etiopathologies (Eid et al., 2019).

Overactivation of the sympathetic nervous system causes up-regulation of SGLT2 pathways. This upregulation increases the risk of vasovagal attacks, myocardial ischemia, cardiac arrhythmias, and sudden death (Kieć-Wilk et al., 2019). SGLT2I therapy could modulate autonomic neuropathy in diabetic patients by regulating the vasovagal tone, heart rate, and sympathetic nervous system. Conversely, the underlying mechanisms of sympathetic changes associated with SGLT2I merit further assessment.

### 4.1 Strengths and Limitations

This meta-analysis compiled the rapidly emerging evidence of the impact of SGLT2I on DPN outcomes. It was conducted systematically following the PRISMA and Cochrane guidelines, limiting potential bias. Furthermore, the meta-analysis included consideration of several outcomes to assess the results of SGLT2I. The comprehensiveness of the meta-analysis notwithstanding, study limitations should be considered when interpreting the meta-analysis results. Significant heterogeneity was detected between the included studies. This heterogeneity might result from differences in patient demographic characteristics, assessment methods, interventions, treatment protocols and follow-up periods. Furthermore, caution is required when interpreting the findings, as many confounders could contribute to the outcomes of SGLT2I administration in patients with DPN.

## 5 Conclusion

SGLT2I could be neuroprotective in patients with DN by considerably increasing the sensory and motor nerve conduction velocity, improving clinical manifestations of DPN, and reducing sympathetic nervous system activity. Integrating these findings into treatment guidelines may help healthcare providers improve DN outcomes in DM patients, satisfying the patient’s desire to adhere to the treatment and enhance their quality of life. However, further studies should be conducted to mitigate the potential limitations of the present meta-analysis.

## Data Availability

The original contributions presented in the study are included in the article/Supplementary Material, further inquiries can be directed to the corresponding author.
